# Transcriptome Sequencing Analysis of the Effects of Metformin on the Regeneration of Planarian *Dugesia japonica*

**DOI:** 10.3390/genes16040365

**Published:** 2025-03-22

**Authors:** Zelong Zhao, Dandan Yin, Kexin Yang, Chunmei Zhang, Linxia Song, Zhenbiao Xu

**Affiliations:** Department of Life Sciences, School of Life and Medicine, West Campus, Shandong University of Technology, Zibo 255000, China; zelong0222@gmail.com (Z.Z.); yindandansdut@163.com (D.Y.); 18632842808@163.com (K.Y.); 19811718781@163.com (C.Z.); slxch@163.com (L.S.)

**Keywords:** metformin, planarian, RNA-seq, *CK1α*, regeneration

## Abstract

Background: Metformin is a widely used oral hypoglycemic agent for treating type 2 diabetes. Planarians, with their remarkable regenerative abilities, are frequently employed as model organisms in stem cell and regeneration studies. This study aimed to investigate the effects of metformin on planarian regeneration, focusing on the regeneration of eyespots after amputation. Methods: Regenerating planarians with amputated eyespots were exposed to various concentrations of metformin. The regeneration time of the eyespots was measured to assess the effects of metformin. Subsequently, a 1 mmol/L metformin treatment for 24 h was applied to the planarians, followed by transcriptome analysis to identify differentially expressed genes (DEGs). The gene expression was validated through qPCR. The full-length gene of casein kinase 1α (*DjCK1α*) was cloned using RACE technology. *DjCK1α* interference was performed to examine its role in regeneration. Results: Low concentrations of metformin significantly reduced the regeneration time of planarians. Transcriptome analysis identified 113 DEGs, including 61 upregulated and 52 downregulated genes. GO and KEGG enrichment analyses were conducted. Notably, *DjCK1α*, a key gene involved in regeneration, was selected for further validation. qPCR confirmed that *DjCK1α* was significantly upregulated. The interference of *DjCK1α* prolonged the regeneration time of the eyespots of planarians cultured in water, while treatment with metformin did not promote the eyespot regeneration of the *DjCK1α*-interfered planarians. Conclusions: The results suggest that metformin accelerates planarian eyespot regeneration, potentially through the regulation of *DjCK1α*. This study provides the first transcriptome-based analysis of drug effects on regeneration in planarians, highlighting the role of metformin in the regeneration process.

## 1. Introduction

Metformin is an oral antidiabetic drug widely used in the treatment of type 2 diabetes mellitus [1,2]. Its main hypoglycemic mechanism is to reduce blood sugar levels by inhibiting liver gluconeogenesis and increasing insulin sensitivity [3,4]. However, an increasing number of in vivo and in vitro studies have found that metformin could not only regulate glucose metabolism but also influence processes such as the cell cycle, aging, apoptosis, proliferation, and regeneration [5,6,7]. Studies by Liao et al. found that 0.1 mmol L^−1^ metformin could promote the proliferation of human umbilical cord mesenchymal stem cells (hUC-MSCs) [8]. Zhang et al. demonstrated that metformin could promote the migration of adult neural precursor cells by upregulating the expression of CDC42 [9]. Fatt et al. showed that metformin promoted neuronal differentiation in female mice by activating the AMPK-APKC-CBP pathway [10]. Xie et al. revealed that metformin accelerated the progress of the regeneration of zebrafish heart by inducing autophagy, enhancing autophagic flux, promoting the regeneration of epicardium, endocardium, and vascular endothelium, accelerating transient collagen deposition and degradation, and inducing the proliferation of cardiomyocytes [11]. The functions of metformin in the processes of cellular biology, especially its effects on cell proliferation and regeneration, have made it a highly promising candidate drug in the field of regenerative medicine [12,13,14].

Planarians, belonging to the phylum Platyhelminthes and class Turbellaria, are widely distributed in freshwater ecosystems such as springs, streams, and lakes around the world [15]. Planarians possess remarkable regenerative capabilities, with pluripotent adult stem cells called neoblasts distributed throughout the body. Neoblasts have the potential to differentiate into cells of any tissue type of planarian, providing a cellular basis for the regeneration [16,17]. After damage to the planarian, neoblasts quickly migrate from various parts of the body to the wound site, where they differentiate into tissues such as neurons, epidermis, and intestine. There are two periods of maximums in mitotic numbers near the wounds in planarians after amputation: one was within 4–12 h and the other was approximately 2–4 days [18]. Planarians have high chemical sensitivity, and many drugs could have effects on them, leading to changes in their morphology, movement, and regenerative ability [19,20]. Therefore, planarians have become one of the model organisms for research on pharmacological and stem cell regeneration [21,22,23,24].

In recent years, transcriptome sequencing technology (RNA-seq) has provided a powerful tool for studying the physiological effects and molecular mechanisms of organisms exposed to chemical drugs [25,26]. It is a high-throughput molecular sequencing technique that can reveal the expression levels of genes under specific conditions by sequencing all the transcripts within cells or tissues, providing the possibility for comprehensive analysis of gene expression [27,28]. The application of this technology can not only reveal differentially expressed genes (DEGs) but also help us understand the functions of these genes in the complex biological processes of organisms, providing profound insights for disease treatment, discovering new therapeutic targets, and understanding cell development and physiological processes [29,30].

In this study, the effects of metformin on the regeneration of the planarian *Dugesia japonica* (*D. japonica*) were investigated using RNA-seq technology. The DEG profiles, Gene Ontology (GO), and Kyoto Encyclopedia of Genes and Genomes (KEGG) pathways were acquired and analyzed to ascertain the genomic responses to the specific stress under metformin exposure. The expression levels of the differential genes *DjCK1α*, *DjCTL* and *DjGPATs* in the transcriptome data were detected using RT-qPCR technology to verify the reliability of the transcriptome sequencing results. To further investigate the role of *DjCK1α* in the regulation of planarian regeneration by metformin, the full length of the *DjCK1α* gene from *D. japonica* was cloned using rapid amplification of cDNA ends (RACE) technology. The effect of metformin on the planarian’s eyespot regeneration was detected after the *DjCK1α* gene was knocked down by RNA interference (RNAi) technology. This study reveals the molecular mechanism by which metformin promotes planarian regeneration and provides a theoretical basis for the further clinical application of metformin.

## 2. Materials and Methods

### 2.1. Materials

The metformin (CAS Number 50-78-2, purity ≥ 99.0%) was purchased from Adamas-beta^®^, Shanghai, China. The TRIzol reagent (Cat. No. 15596) was purchased from Invitrogen Corporation, Waltham, MA, USA. The NEBNext^®^ Ultra^TM^ RNA Library Prep Kit (NEB #E7530L), Oligo d (T) Magnetic Beads (NEB #S1419S), Fragmentation Buffer (NEB #E6150S), M-MuLV reverse transcriptase (NEB #M0253S) and RNase H (NEB #M0297S) were purchased from New England Biolabs, England. The AMPure XP beads (Cat. No.A63880) were purchased from Beckman Coulter, America. The Reverse Transcription Kit (Code No. 6215A), TB Green premix Ex Taq II (Code No. RR420A), IPTG (Code No. 9030), *Pst I* (Code No. 1073A), *Xho I* (Code No. 1094A), and SMARTer RACE 5/3 Kit (Code No. 634859) were purchased from Takara Bio Inc, Japan. The plasmids pUC19 and L4440, as well as *E. coli* HT115, were preserved in our laboratory.

### 2.2. Experimental Animals

The planarian used in this study was an asexual strain Dugesia ZB-1 obtained through cutting and cultivated in the laboratory. The planarians were cultured in a biochemical incubator (SPX-2508SH, Shanghai CIMO Medical Instrument Manufacturing Co., Ltd., Shanghai, China) at 20 °C in Montjuïc water (1.6 mmol L^−1^ NaCl, 1.0 mmol L^−1^ CaCl_2_, 1.0 mmol L^−1^ MgSO_4_, 0.1 mmol L^−1^ MgCl_2_, 0.1 mmol L^−1^ KCl, and 1.2 mmol L^−1^ NaHCO_3_). They were fed with nematode homogenate once a week and subjected to starvation for one week before the experiments.

### 2.3. Effect of Metformin Exposure on Planarian Eyespot Regeneration

**Figure 1:**
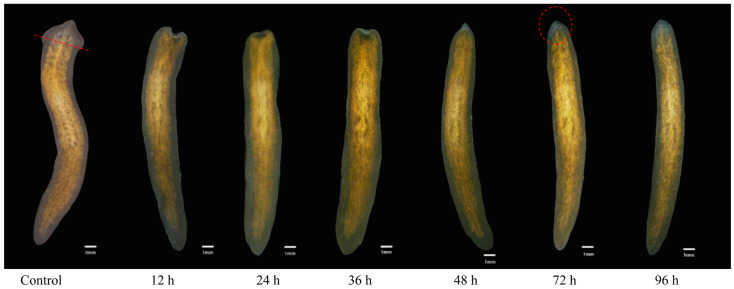
Regeneration process of eyespots in planarians. The red line represents the cutting site.

Planarians of approximately the same size were selected for the experiment and transverse amputation was performed along the posterior edge of the planarian eyespots (Figure 1). Fragments of planarians lacking eyespots were exposed to clean water and metformin at concentrations of 0.1 nmol L^−1^, 1 nmol L^−1^, 10 nmol L^−1^, 100 nmol L^−1^, 1 µmol L^−1^, 10 µmol L^−1^, 1 mmol L^−1^, 10 mmol L^−1^, and 40 mmol L^−1^, respectively. Through preliminary experiments, we found that it took more than 60 h for the eyespots of the planarians to complete regeneration after amputation. Therefore, we took pictures and observed every 12 h within the first 60 h, and every 1 h after 60 h. When two distinct and symmetrical black eyespots could be clearly seen under a stereomicroscope, the time was recorded as the eyespot regeneration time. Each treatment contained 30 planarians, and the regeneration time of planarian eyespots was observed and recorded. The experiment was repeated three times.

### 2.4. Construction and Sequencing of High-Throughput Transcriptome Library

A total of 100 regenerating planarians with cut eyespots were separately exposed to clean water and 1 mmol L^−1^ metformin solution for 24 h. The total RNAs were extracted from the planarians. The purity of the RNA samples was determined using a NanoPhotometer NP80 (Implen GmbH, Munich, Germany) by measuring the OD_260_/OD_280_. The RNA integrity was detected using an Agilent 2100 Bioanalyzer (Agilent Technologies, Santa Clara, CA, USA) and its concentration was quantified using a Qubit 2.0 Fluorometer (Thermo Fisher Scientific, Waltham, MA, USA). The NEBNext^®^ Ultra^TM^ RNA Library Prep Kit for Illumina^®^ and the total RNA, with a minimum quantity of more than 1 µg, was used for library construction. The mRNAs with polyA tails were enriched using oligo d(T) magnetic beads, followed by fragmentation of the mRNAs into small pieces using divalent cations in the fragmentation buffer. The fragmented mRNAs were then used as templates for the first-strand cDNA synthesis with random hexamer primers in the presence of M-MuLV reverse transcriptase, followed by degradation of the RNA strands with RNase H and synthesis of the second cDNA strand using dNTPs as substrates in the DNA polymerase I system. The purified double-stranded cDNAs were subjected to end repair, A-tailing, and adapter ligation, followed by size selection of approximately 200 bp cDNA fragments using AMPure XP beads. PCR amplification was performed with the size-selected cDNAs as templates, and the products were purified using AMPure XP beads to obtain the final library. The library was quantified using a Qubit 2.0 Fluorometer and diluted to the concentration of 1.5 ng μL^−1^. The insert size of the library was assessed using an Agilent 2100 Bioanalyzer and the effective concentration of the library was quantified using qRT-PCR. The effective concentration had to be more than 2 nmol L^−1^ to ensure the quality of the library. The libraries were pooled according to their effective concentrations and subjected to Illumina sequencing.

### 2.5. Bioinformatics Analysis of Transcriptome Sequencing

The raw data were filtered using fastp v0.19.3 software to remove reads containing adapter sequences. Paired reads were discarded if the N content exceeded 10% of the bases in either read, or if the proportion of low-quality bases (Q ≤ 20) exceeded 50% of the bases in either read. All the analyses were conducted based on the resulting clean reads. The clean reads were assembled into transcripts using Trinity v2.11.0 software. The assembled transcripts were clustered and redundant transcripts were removed using Corset version 1.09 software. Statistical power for the experiment was calculated using RNASeqPower 1.44.0 software. In order to identify the DEGs between the two different samples, the expression level for each transcript was calculated using the fragments per kilobase of exon according to the million mapped reads (FRKM) method. DESeq2 1.22.2 was used for the differential expression analysis. To understand the functions of the DEGs, GO functional enrichment and KEGG pathway analysis were carried out by Goatools and KOBAS. The DEGs were significantly enriched in the GO terms and metabolic pathways when their Bonferroni-corrected *p*-value was less than 0.05.

### 2.6. Validation of Transcriptome Sequencing Using qPCR

Three DEGs, casein kinase 1 alpha (*DjCK1α*), C-type lectin (*DjCTL*), and glycerol-3-phosphoacyl transferase (*DjGPATs*), were selected for validation of the RNA-seq results. Planarians with cut eyespots were treated with 1 mmol L^−1^ metformin for 24 h, and those cultured in clean water served as controls. The total RNAs were extracted using TRIzol reagent from both groups and reverse transcribed into cDNAs. The reverse transcription system was 20 μL containing 2 μg RNA. The primers for the qPCR are listed in Table A1, and they were designed using Primer 5.0 software with *Djactin* as the internal reference. The relative expression change of each mRNA was analyzed using the 2^−ΔΔCt^ method. The qPCR reactions were performed using a real-time fluorescence quantitative PCR instrument (Roche Light Cycler 480) with three replicates per group.

### 2.7. Cloning of DjCK1α Gene Using RACE

The total RNA was extracted using the TRIzol reagent, and the oligo (dT) primer was used for reverse transcription. The RACE experiment was conducted according to the manual for the RACE kit. The primer sequences are shown in Table A2. The amplified 3′ fragment and 5′ fragment of *DjCK1α* were cloned into pUC19 and sequenced to obtain the full-length sequence information of the *DjCK1α* gene. The bioinformatics analysis was performed on the NCBI Web server (http://www.ncbi.nlm.nih.gov/blast, accessed on 1 June 2024). The open reading frame (ORF) was predicted using the ExPASy server (http://web.expasy.org/translate, accessed on 2 June 2024), and the multiple sequence alignment was performed using GeneDoc 2.7.000 software.

### 2.8. RNA Interference (RNAi)

The primers used for the interference of the *DjCK1α* gene were designed with Primer 5.0 software, and they are shown in Table A3. The total RNA of the planarians was extracted and reverse transcribed into cDNA. The amplified fragment of the *DjCK1α* gene and the plasmid L4440 were digested with *PstI* and *XhoI* and then ligated. The recombinant interference vector *L4440-DjCK1α* was transformed into the competent cell HT115 and expression was induced with 1 mmol L^−1^ IPTG. The expressed bacterial cells were mixed with nematode homogenate in a 1:2 weight ratio and fed continuously to the planarians for four days. The planarians fed with expressed bacteria of the empty vector L4440 and nematode homogenate were used as controls. On the 5th day, the total RNAs of the planarians were extracted and reverse transcribed into cDNA, and qPCR was performed to detect the interference efficiency.

### 2.9. Data Analysis

Statistical analysis was performed using SPSS 16.0 and GraphPad Prism 8.0. Data for the eyespot regeneration and gene expression levels are presented as the mean ± SD. Differences between the treatment and control groups were first assessed using one-way ANOVA. If significant differences were found (*p* < 0.05), post hoc pairwise comparisons were performed using *T*-tests to determine which groups differed significantly. A value of *p* < 0.05 was considered statistically significant and *p* < 0.01 as highly significant.

## 3. Results

### 3.1. Effect of Metformin Exposure on the Regeneration of Eyespots in Planarian

**Figure 2:**
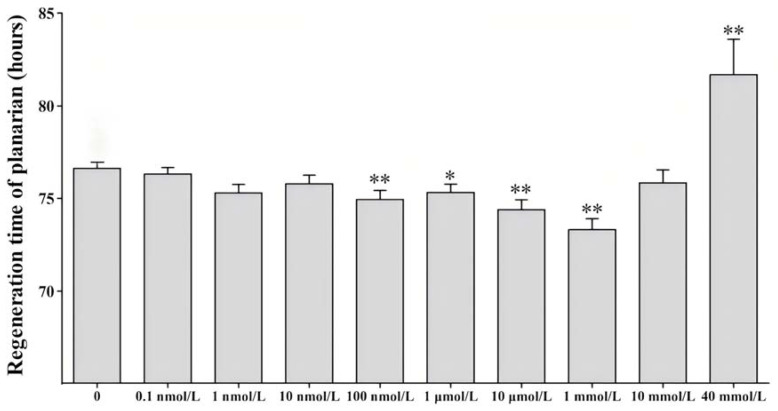
The regeneration time of planarian eyespots treated with metformin. Here, 0 represents the regeneration time of the eyespots of planarians cultured in clean water, while the others represent the regeneration time of the eyespots of planarians treated with different concentrations of metformin (* *p* < 0.05; ** *p* < 0.01).

After transverse amputation, the planarians were exposed to clean water and metformin at concentrations of 0.1 nmol L^−1^, 1 nmol L^−1^, 10 nmol L^−1^, 100 nmol L^−1^, 1 µmol L^−1^, 10 µmol L^−1^, 1 mmol L^−1^, 10 mmol L^−1^, and 40 mmol L^−1^. The regeneration time of the planarian eyespots was 76.57 h, 76.25 h, 75.25 h, 75.75 h, 74.9 h, 75.27 h, 74.35 h, 73.27 h, 75.8 h, and 81.65 h, respectively (Figure 2). Among these concentrations, metformin at 100 nmol L^−1^, 10 µmol L^−1^, and 1 mmol L^−1^ could significantly shorten the regeneration time of planarian eyespots, and 1 mmol L^−1^ had the best facilitation effect, while 40 mmol L^−1^ had the greatest inhibition effect on the regeneration process. Therefore, we chose 1 mmol L^−1^ metformin to treat the planarians for 24 h and then performed the subsequent transcriptome sequencing analysis.

### 3.2. High-Throughput Transcriptome Analysis

**Figure 3:**
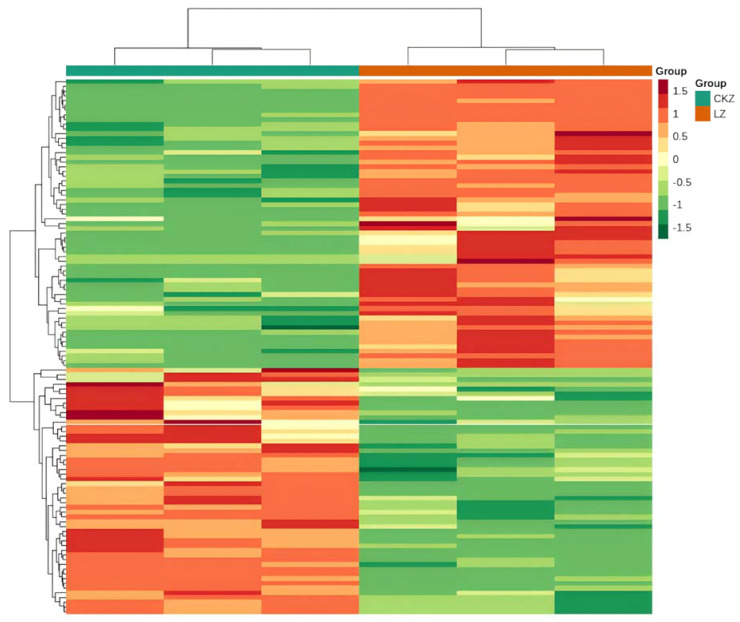
Differentially expressed gene analysis in metformin−exposed planarians. Hierarchical clustering of differentially expressed genes based on the TPM values. The color from red to green denotes gene expression levels from high to low. CKZ represents the control group and LZ represents the metformin-exposed group. Three replicates are for each sample. Regeneration time of planarian eyespots treated with metformin.

**Figure 4:**
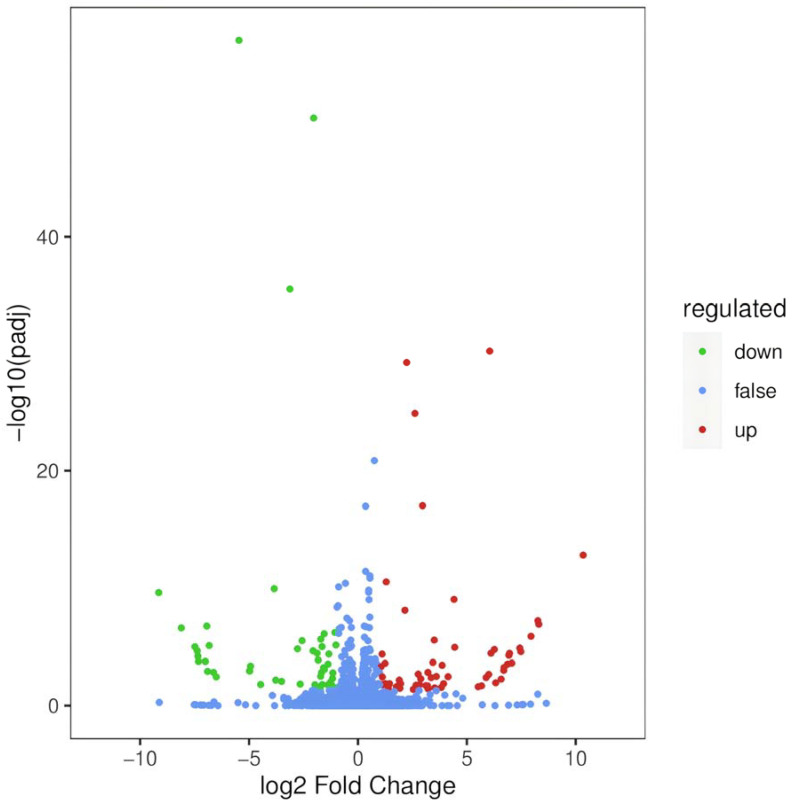
Volcano plot of the gene libraries of planarians between the metformin-treated group. Differences in gene expression are showed in terms of the fold change (FC) and significance (*p* value). Each dot represents a gene: the red dot represents upregulated differentially expressed genes, the green dot represents downregulated differentially expressed genes, and the blue dot represents genes without differential expression.

**Figure A1:**
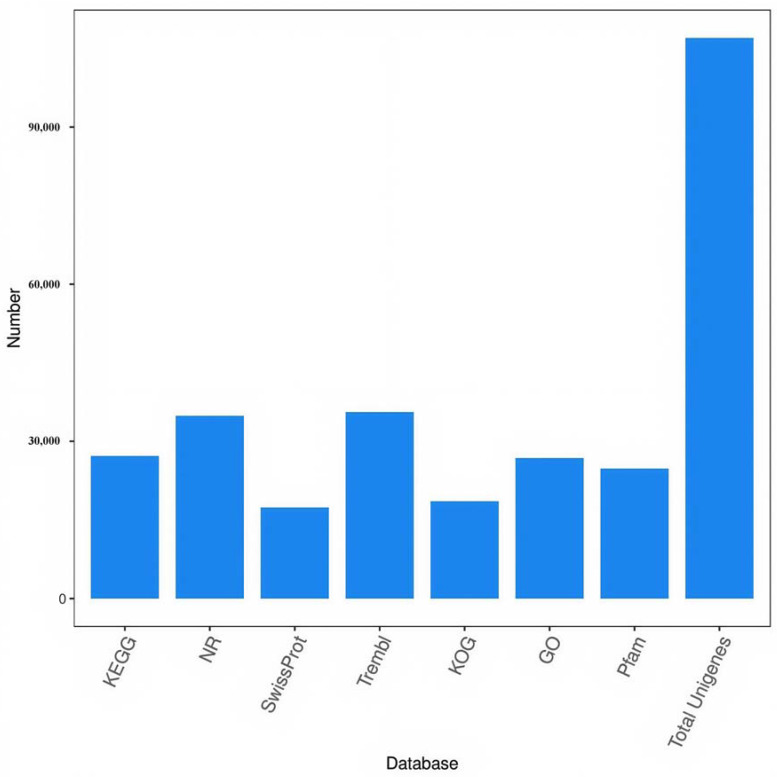
Statistics diagram of the number and annotation of the unigenes. The horizontal axis represents the names of different databases, and the vertical axis represents the number of unigenes annotated in different databases and the total number of unigenes.

RNA extraction was conducted using the regenerating planarians treated with 1 mmol L^−1^ metformin for 24 h, and those cultured in clean water served as controls. Then, the cDNA library was constructed and the transcriptome sequencing was performed. As shown in Table A4, compared with the control group, there was a significant change in the gene expression profile of the regenerating planarians after 24 h of exposure to 1 mmol L^−1^ metformin. It produced approximately 265,936,166 total clean reads from the 39.9 Gbp clean bases data for all the biological replicates, and more than 89% of the clean reads had quality scores over the Q30 value, The statistical power of this experimental design, calculated in RNASeqPower, was 0.81. The expression quantity of each gene was normalized to FPKM to compare among different samples. Hierarchical clustering of representative unigenes of six samples suggested that differential gene expression appeared in metformin-treated planarians (Figure 3). A volcano plot of the DEGs was constructed based on the *p*-values and folding changes of each transcript (Figure 4), with the standard settings of a *p*-value < 0.05 and absolute log2 (fold change) > 1. The assembled 98,398 unigenes were compared against multiple databases. NR, PFAM, Trembl, and Swiss-Prot annotated the protein sequences, while KOG, GO, and KEGG annotated the gene functions, along with the related biological processes and metabolic pathways. The gene numbers in each database and the total are shown in Figure A1. Genes annotated in at least one database made up 37.36% of the total, suggesting many unknown genes in *D. japonica* await study. The results showed that there were 113 genes differentially expressed after treatment with 1 mmol L^−1^ metformin in the regenerating planarians, including 52 downregulated and 61 upregulated genes.

**Figure 5:**
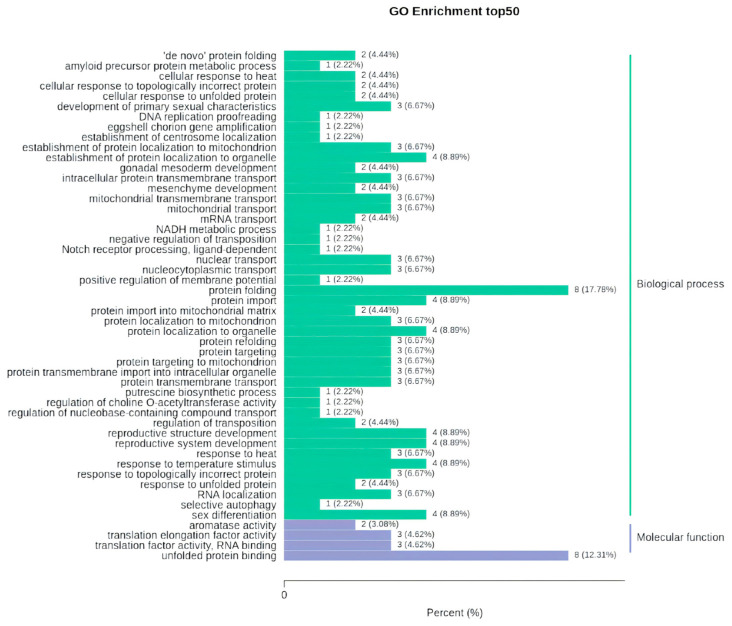
GO enrichment of differentially expressed genes. The horizontal axis represents the proportion of annotated genes to the total number of annotated genes, while the vertical axis represents the name of the GO entry. The label on the right side of the chart indicates the category to which the GO entry belongs.

**Figure A2:**
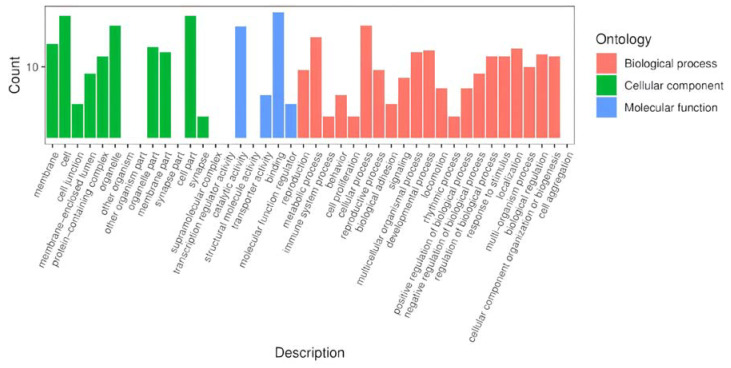
GO classification diagram of differentially expressed genes. The horizontal axis represents the secondary GO entries, and the vertical axis represents the proportion of differentially expressed genes to the total number of differentially expressed genes. The distribution of the GO categories was assigned to three categories: biological process, cellular component, and molecular function.

GO classification annotation and enrichment analysis were conducted on the 113 DEGs, of which 89 were annotated in the GO database and were enriched in three major categories: biological processes, cellular components, and molecular functions. The annotation classification results are illustrated in Figure A2. The basic GO unit is the GO term, and each GO term belongs to one of the specific categories. The top 50 GO term entries with the lowest q-value in the enrichment analysis were selected and are displayed in Figure 5, where the pathways of protein folding and unfolded protein binding have the most significantly enriched differential genes.

**Figure 6:**
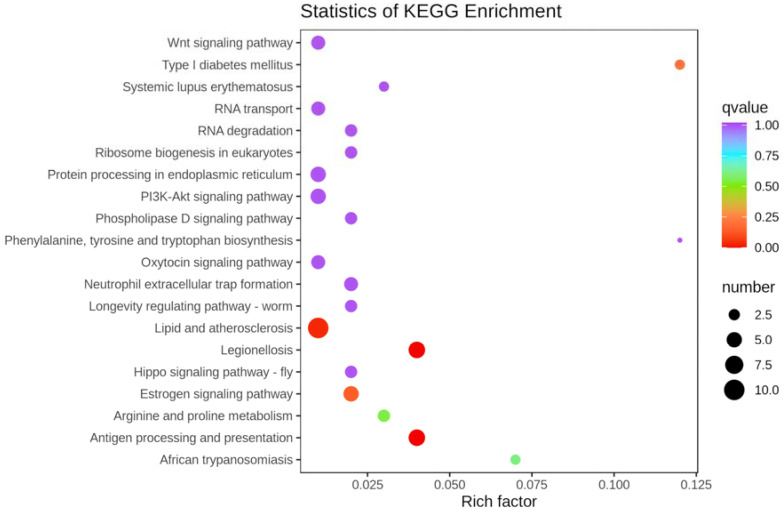
Statistics of the KEGG enrichment. The y-axis represents the KEGG pathway, and the *x*-axis represents the Rich factor. The higher the Rich factor, the higher the degree of enrichment. The size of the dot represents the number of differentially expressed genes enriched in the pathway. The color of the dot indicates the significance of the enrichment, with red indicating the highest significance.

The 113 DEGs were subjected to KEGG annotation classification and enrichment analysis, and the results showed that 91 genes were annotated in the KEGG database, involving a total of 189 signaling pathways. According to their involvement in metabolic pathways, these genes can be classified into six major categories based on their functions, namely cellular processes, containing 16 signaling pathways; environmental information processing, containing 26 signaling pathways; genetic information processing, containing 10 signaling pathways; human diseases, containing 55 signaling pathways; metabolism, containing 21 signaling pathways; and organismal systems, containing 61 signaling pathways. Figure 6 shows the top 20 up- or downregulated KEGG pathways based on the *p*-values. Among these pathways, the following three related to immune response and anti-inflammatory were significantly enriched (*p* < 0.05), with lipid and atherosclerosis (ko05417) being the most enriched, followed by legionellosis (ko05134) and antigen processing and presentation (ko05417).

### 3.3. Validation of Transcriptome Sequencing by qPCR

**Figure A3:**
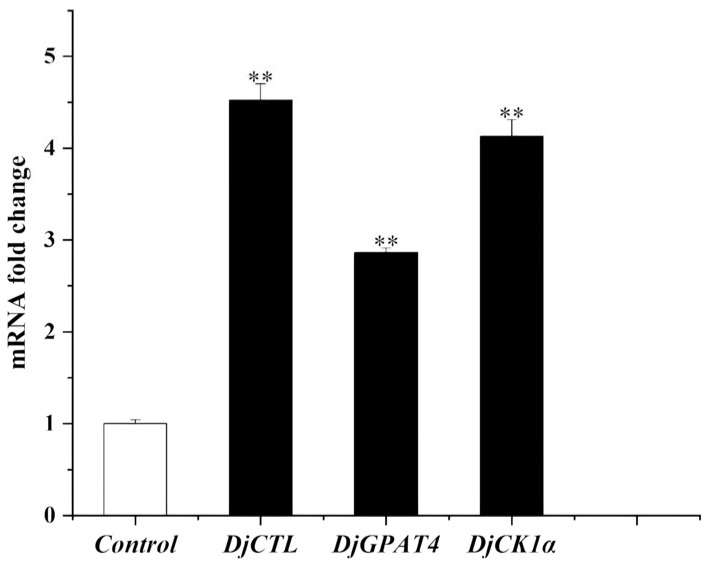
Validation of the transcriptome sequencing data. The mRNA expression levels of the *DjCTL*, *DjGPAT4*, and *DjCK1α* genes after 1 mmol L^−1^ metformin exposure for 24 h. Standard deviation is represented by the error bars. Each experiment was repeated three times. Asterisk indicates statistical differences (** *p* < 0.01).

In order to verify the reliability of the transcriptome data, three significantly upregulated genes, casein kinase *DjCK1α*, C-type lectin *DjCTL* and glycerol-3-phosphate acyltransferase *DjGPAT4*, were selected for validation using qPCR. The total RNAs were extracted from the planarians treated with 1 mmol L^−1^ metformin for 24 h, and those cultured in clean water served as controls. After reverse transcription, the cDNAs were subjected to qPCR for validation. The results are shown in Figure A3. The expression levels of *DjCTL*, *DjGPAT4*, and *DjCK1α* in the metformin-treated planarians were significantly upregulated, indicating that the results from the transcriptome database are reliable.

### 3.4. Cloning of Planarian DjCK1α by RACE

**Figure 7:**
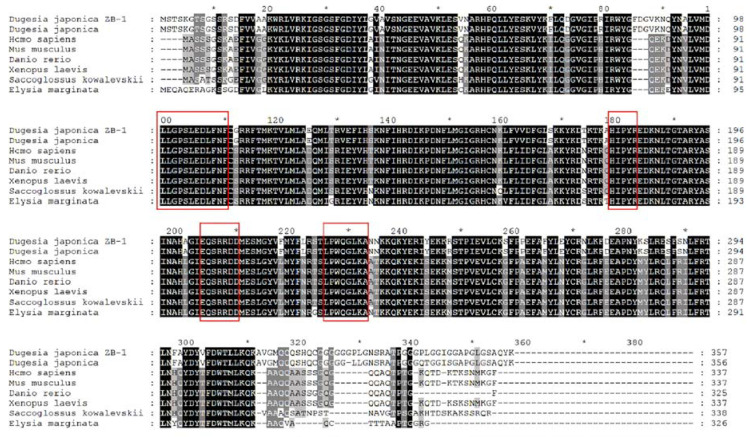
Multi-sequence alignment of *Dugesia japonica DjCK1α* and the casein kinase 1 of other species. Species and sequence ID: *Dugesia japonica ZB-1* (PP871396), *Dugesia japonica* (WFL37650.1), *Homo sapiens* (NP_001020276.1), *Mus musculus* (NP_001344427.1), *Danio rerio* (XP_009289364.1), *Xenopus laevis* (NP_001079933.1), *Saccoglossus kowalevskii* (NP_001158490.1), *Elysia marginata* (GFR90168.1). “*” represents completely conserved sites.

**Figure A4:**
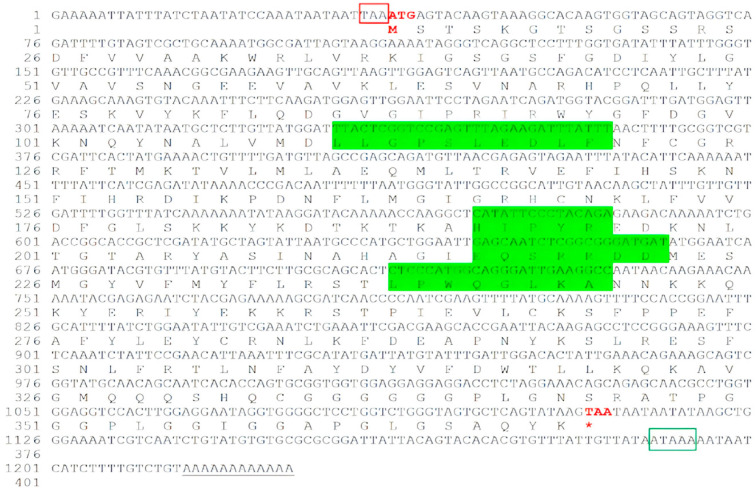
Nucleotide and deduced amino acid sequence of DjCK1α. The initiator and terminator codons are marked in the red and bold font. The conserved characteristic sequences LLGPSLEDLF, HIPXR, EXSRRDD, and LPWQGLKA of casein kinase 1 are highlighted in green. The tail addition recognition signal AATAAA is boxed in green and the poly A tails are double underlined. There are two stop codons (TAA) boxed in red in front of the start codon ATG. “*” represents a stop codon.

Among the three upregulated genes mentioned above, we chose the *DjCK1α* gene for further research. The full-length *DjCK1α* gene was cloned using RACE technology. Bioinformatics analysis showed that the full-length of *DjCK1α* is 1226 bp, with an open reading frame of 1074 bp, which encodes 357 amino acids (Figure A4). Its molecular weight is approximately 40.29 kDa, and the theoretical isoelectric point is 8.931. The gene sequence exhibits the highest homology with the *D. japonica* casein kinase I isoform alpha (OP379657.1) published by Huang et al., with a gene homology of 92.37% and a protein homology of 98.60% [31]. Multiple sequence alignment showed that the amino acid sequence encoded by *DjCK1α* is highly conserved compared to that of other species (Figure 7). It contains the characteristic sequences LLGPSLEDLF, HIPXR, EXSRRDD, and LPWQGLKA.

### 3.5. Functional Study of DjCK1α in Metformin-Regulated Eyespot Regeneration in Planarians

**Figure A5:**
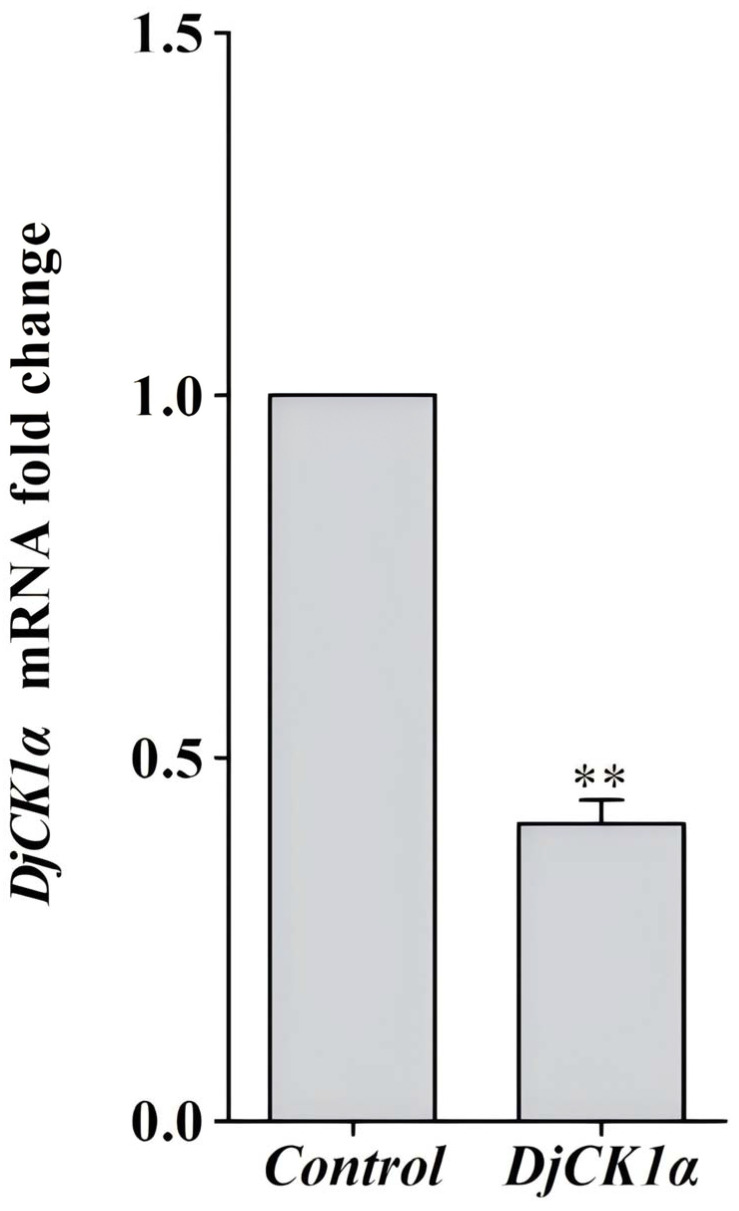
Detection of *DjCK1α* RNAi efficiency. The mRNA expression levels of the *DjCK1α* genes after RNAi. Standard deviation is represented by the error bars. (** *p* < 0.01).

In order to investigate the role of the *DjCK1α* gene in metformin-regulated eyespot regeneration, planarians of approximately the same size were selected for the interference of the *DjCK1α* gene. They were fed with bacteria food mixture of the recombinant plasmid containing the *DjCK1α* gene, and the bacteria food mixture containing the empty vector was used as a control. After continuous feeding for 4 days, the total RNAs were extracted and reverse transcribed into cDNA. The interference efficiency was detected by qPCR and the result is shown in Figure A5. The interference efficiency of the *DjCK1α* gene was 60%.

**Figure 8:**
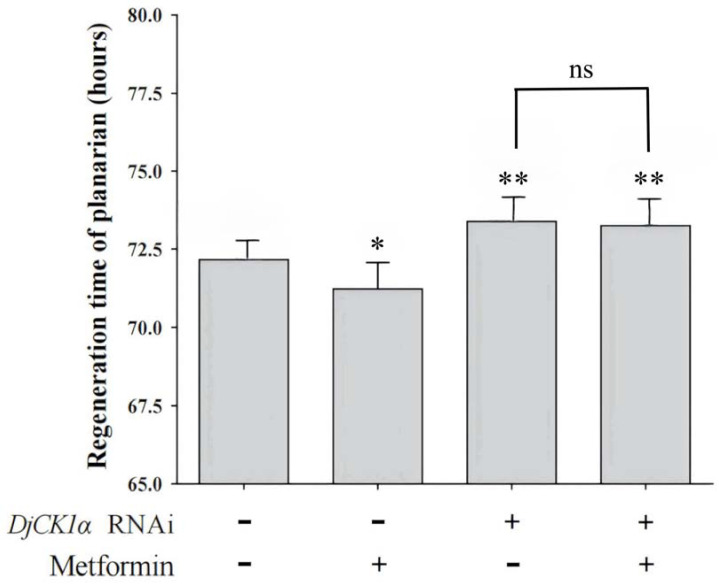
Regeneration time of planarian eyespots. Regeneration time of the eyespots in planarians after co-treatment with metformin and DjCK1α-RNAi. Standard deviation is represented by the error bars. Asterisk indicates statistical differences (* *p* < 0.05; ** *p* < 0.01, ns represents no significant difference).

The eyespots of the planarians were cut after interference and the regenerating planarians were cultured in clean water and 1 mmol L^−1^ metformin solution, respectively. Each group contained 30 planarians. The regeneration time of the eyespots of the planarians was observed and recorded. The experiments were divided into the following four groups: non-interfered planarians cultured in water, interfered group cultured in water, non-interfered planarians treated with metformin and interfered group treated with metformin. The eyespot regeneration times of the groups were compared and analyzed. The result is shown in Figure 8. The eyespot regeneration times of the four groups were 72.15 h, 73.38 h, 71.23 h, and 73.25 h, respectively. Compared with the non-interfered group cultured in water, the eyespot regeneration time of the interfered group cultured in water was significantly prolonged, indicating that *DjCK1α* was involved in the regeneration process of eyespots and the interference of this gene inhibited the eyespot regeneration in planarians. There was no significant difference in the eyespot regeneration time between the interfered group treated with metformin and the non-interfered group treated with metformin, indicating that metformin could not promote the regeneration of eyespots in planarians after the interference of *DjCK1α*, suggesting that metformin might regulate the regeneration of eyespots in planarians through the action of *DjCK1α*.

## 4. Discussion

Wound healing and tissue regeneration are crucial for the normal survival of organisms. Many organisms have the ability to regenerate, and the abilities of different organisms or organs of the same species are different [32]. In the human body, some organs have the ability to regenerate, such as the skin repair and the liver regeneration after injury [33,34]. However, the regenerative abilities of these organs are limited. Once severely damaged, it is difficult for them to complete regeneration [35]. Therefore, revealing the regeneration mechanism of species with complete systemic regeneration ability is of great significance for studying the regeneration of human tissues and organs. In nature, some invertebrates, such as sponges, hydras, and planarians, have the ability to regenerate the whole body [36,37,38]. Due to their anatomical structure, genome, and position in the evolutionary tree, planarians have been increasingly chosen as the model organism for the study of regeneration [39,40]. In recent years, with the development of biochemical and molecular biology technologies, the research on planarian regeneration has mainly focused on discovering and identifying key genes that regulate regeneration, molecular mechanisms that regulate the fate of adult pluripotent stem cells, and molecular mechanisms that regulate polarity reconstruction during regeneration [41,42,43]. However, there are no relevant reports on the regulatory effect of chemical drugs on the regeneration of planarians. Metformin is the first-line drug for the treatment of type 2 diabetes in clinical medicine. It can reduce blood sugar by inhibiting the production of liver glucose. In recent years, a large number of studies have shown that metformin could affect the differentiation of stem cells, improve their immune regulatory properties, and exert the anti-aging, antioxidant, and anti-inflammatory effects of stem cells, thus affecting the regeneration process of damaged tissues or organs [44,45,46,47]. Given the extensive pharmacological effects of metformin, we used different concentrations of metformin to treat the regenerating planarians after the amputation of their eyespots. We found that low concentrations of metformin could promote the regeneration of planarian eyespots, while high concentrations of metformin could inhibit the regeneration. Therefore, we conducted transcriptome analysis on planarians treated with 1 mmol L^−1^ metformin to explore the mechanism of metformin in regulating eyespot regeneration in planarians.

Transcriptome analysis is a technique used to study gene expression by measuring the levels of RNA molecules [48]. It can help researchers understand information on gene expression patterns, regulatory mechanisms, cellular signaling pathways, and the mechanisms of disease occurrence in cells. Although changes in mRNA expression do not always correlate directly with changes in the corresponding protein levels, they still provides valuable insights for inferring specific changes in organisms [49]. So far, RNA-seq has been used in research fields such as regeneration, development, and immunity in planarians [50,51,52]. To investigate the regulatory mechanisms of low-concentration metformin on planarian eyespot regeneration, we performed transcriptome sequencing on regenerating planarians after the amputation of eyespots and exposure to 1 mmol L^−1^ metformin solution for 24 h. From six sequencing libraries, approximately 6.24~7.00 Gb of clean reads were obtained and assembled into 107,011 single genes with an average length of 796 bp. The number of single genes obtained in our study exceeded that assembled by Qin et al. from the transcriptome analysis during planarian regeneration (37,218), and the average length of the single genes we assembled (796 bp) was much longer than that of their transcriptome single genes (468 bp) [50]. Species distribution analysis suggested that the majority of unigenes showed homology to the freshwater planarian *Schmidtea mediterranea* and other Platyhelminthes species, indicating the reliability of the sequencing and assembly. Notably, a significant portion of unigenes could be functionally assigned to a wide range of GO categories, indicating the inclusion of diverse transcripts related to numerous biological processes in the sequence data. Moreover, most of the representative unigenes were annotated to specific pathways, such as signal transduction, transport and catabolism, translation, as well as amino acid metabolism, suggesting that the majority of the genes we identified were involved in the signal transduction and physiological processes of *D. japonica* in response to metformin. These results indicate that our sequencing coverage is deep enough and the assembly quality is high enough for subsequent functional analyses.

Through transcriptome data analysis, we found that there were 113 DEGs between the 1 mmol L^−1^ metformin-treated group and the control group in regenerating planarians, with 61 upregulated genes and 52 downregulated genes. Different gene products in organisms exert biological functions through interactions with each other, and pathway annotation analysis of DEGs helps to elucidate gene functions. GO functional enrichment analysis of the DEGs showed that transcripts related to DNA damage repair, stress response, and cell apoptosis were the main components. KEGG pathway enrichment analysis suggested that the metabolism and signal pathways involved in immune response, detoxification, and inflammation were significantly enriched. Recently, an increasing number of pharmacological studies in various animals have shown that GO terms and metabolic pathways related to immunity, detoxification, antioxidant, DNA repair, and apoptosis are significantly enriched under drug treatment [53,54,55]. Therefore, our study indicates that treatment with metformin leads to significant changes in several metabolic and stress-related pathways in planarians. However, we also note that some of these signaling pathways are uncommon in invertebrates, suggesting that further experiments are needed to verify their biological significance.

Casein kinase 1 (CK1) is an ancient and conserved multifunctional protein, which is widely distributed in eukaryotes, primarily found in the nucleus, cytoplasm, cell cytoskeleton, and cell membrane. It is involved in various physiological activities, such as cell division, apoptosis, DNA repair, p53 regulation, and circadian rhythms, as well as the occurrence and development of tumors [56,57,58]. CK1 participates in signaling pathways, including Wnt, Hedgehog, Hippo, and NF-κB, regulating cellular proliferation, embryonic development, immune responses, energy metabolism, and circadian rhythms [59,60,61,62]. Penas et al. showed that the expression level of *CK1* significantly increased during the proliferation and division of cerebellar granule cell progenitors (GCPs), and the upregulation or degradation inhibition of *CK1* might be related to the stimulation of neuronal neurite regeneration [63]. Magliozzi et al. found in mouse epithelial cells that CK1 and I-kappa-B-kinase-β could synergistically phosphorylate the Rap guanine exchange factor (RAPGEF2), thereby regulating the development and regeneration of mouse epithelial tissue [64]. Metformin directly acts on the liver, kidneys, and intestines, and it primarily exerts pharmacological effects such as reducing the fat content and lowering the blood sugar by activating the AMP-activated protein kinase (AMPK) signaling pathway [65,66,67,68]. Jee Hyun et al. demonstrated that phosphorylation of the Ser-389 site of rat CK1 by the AMPK signaling pathway could lead to an increase in CK1 activity [69]. In our study, the expression of the *DjCK1α* gene was significantly upregulated in the regenerating planarians after exposure to 1 mmol L^−1^ metformin. In order to further investigate whether *DjCK1α* is involved in the eyespot regeneration process of metformin-regulated planarians, we cloned the full-length planarian *DjCK1α* gene by RACE technology and performed bioinformatics analysis. The results of the protein and nucleic acid sequence alignments showed that it differs from the published sequences of *D. japonica*, suggesting it may be another isoform of *DjCK1α* [31]. The interference vector L4440-*DjCK1α* was constructed and the expressed bacteria was fed to planarians to downregulate the expression of *DjCK1α*, and then the eyespots of the interfered planarians were cut and the time of regeneration was recorded. The results showed that after the interference of *DjCK1α*, the regeneration time of the eyespots was prolonged, while treatment with metformin did not promote the eyespot regeneration of the *DjCK1α*-interfered planarians, indicating that metformin might regulate the regeneration of eyespots in planarians through *DjCK1α*.

In recent years, stem cells have become a research hotspot in the fields of biology and medicine, having been used in the field of regenerative medicine, such as treating autoimmune diseases, neurological diseases, tissue damage, and cardiovascular diseases [70,71,72,73]. The strong regenerative ability of planarians is due to their pluripotent stem cells throughout the body; therefore, studying the regenerative mechanism of drugs in planarians is of great significance. In this study, transcriptome sequencing analysis was conducted using regenerating planarians with cut eyespots and treatment with 1 mmol L^−1^ metformin for 24 h. This is the first report on the correlation between gene transcription changes and biological network changes during planarian regeneration after exposure to metformin. This work provides global and specific information about the adaptive response of planarians to the effects of metformin. Due to the multifunctionality of CK1 and its potential as an attractive drug target, we cloned the full-length *DjCK1α* gene using RACE technology and conducted bioinformatics analysis. Combined with RNAi technology, we demonstrated that the *DjCK1α* gene may be one of the potential targets regulated by metformin during planarian eyespot regeneration. This study provides not only a theoretical basis for investigating the molecular mechanism of metformin promoting planarian regeneration but also a reference for the application of metformin in the field of stem cell regeneration.

## 5. Conclusions

This study explored the effects of metformin on eyespot regeneration in planarians. We found that 1 mmol L^−1^ metformin significantly shortened the regeneration time, while 40 mmol L^−1^ inhibited it. Transcriptome analysis revealed 113 differentially expressed genes in the metformin-treated planarians, with significant involvement in cellular processes and immune responses. qPCR validation showed that *DjCK1α*, *DjCTL*, and *DjGPAT4* were upregulated, confirming the transcriptome data. Functional studies on *DjCK1α* indicated that interference with this gene delayed eyespot regeneration, and metformin could not reverse this delay, suggesting that metformin regulates regeneration through *DjCK1α*. This study demonstrates that metformin influences eyespot regeneration in planarians, with *DjCK1α* playing a crucial role in mediating these effects.

## Data Availability

The raw gene sequences have been uploaded to the NCBI database with the accession number PP871396. The sequences can be accessed on 4 June 2024 at the following link: https://www.ncbi.nlm.nih.gov/nuccore/PP871396.1/.

## Appendix A

**Table A1:** The primers for qPCR.

Primer Name	Sequence (5’-3’)
DjCTL-qPCR-F	TCTTCAGGTTCCCAAGGTCT
DjCTL-qPCR-R	CTGATTGGAAGAGTAGTGATGGT
DjCK1α-qPCR-F	ATTTGGGTGTTGCCGTTTC
DjCK1α-qPCR-R	CGGACCGAGTAAATCCATAAC
DjGPAT4-qPCR-F	CATCGGATTCTACACCTTTTTGC
DjGPAT4-qPCR-R	GAAACTTTGCCACATCCACCA

**Table A2:** The primers for RACE.

Primer Name	Sequence (5’-3’)
5R-DjCK1α-1	GATTACGCCAAGCTTGCCTTCAATCCCTGCCATGG
3R-DjCK1α-1	GATTACGCCAAGCTTGATGTTAGCCGAGCAGATGTT
5R-DjCK1α-2	GAAGACAAAAATCTGACCGGC
3R-DjCK1α-2	CTATTTGTTGTTGATTTTGGTTTAT
UPM short primer	CTAATACGACTCACTATAGGGC

**Table A3:** The primers for RNAi.

Primer Name	Sequence (5’-3’)
DjCK1α-RNAi-F	AACTGCAGGATGTTAGCCGAGCAGATGTT
DjCK1α-RNAi-R	CCGCTCGAGCCCCACCTATTCCTCCAAG

**Table A4:** Quality statistics of the clean reads.

Samples	Raw Reads	Clean Reads	Clean Base (G)	Q30 (%)	GC (%)
CK1	48,759,412	46,671,068	7	91.09	33.83
CK2	43,642,502	41,630,244	6.24	91.84	33.58
CK3	44,409,026	42,310,516	6.35	90.48	33.77
L1	48,116,106	45,890,448	6.88	90.42	33.78
L2	44,956,770	42,980,480	6.45	89.99	33.72
L3	48,757,002	46,453,410	6.97	90.7	33.84
